# Effect of High-Pressure Processing on Storage Quality, Bioactive Compounds, and Flavor Profile of Selenium-Sand Melon Juice

**DOI:** 10.3390/foods15183292

**Published:** 2026-09-17

**Authors:** Li-Li Li, Zhi-Jing Ni, Run-Hui Ma, Wei Wang, Kiran Thakur, Zhao-Jun Wei

**Affiliations:** 1 School of Biological Science and Engineering, Specialty Food Nutrition and Health Innovation Team of Ningxia Hui Autonomous Region, North Minzu University, Yinchuan 750021, China; 2 Ningxia Key Laboratory for the Development and Application of Microbial Resources in Extreme Environments, Yinchuan 750021, China

**Keywords:** *Citrullus lanatus*, non-thermal processing, microbial inactivation, flavor compounds, non-thermal sterilization

## Abstract

This study systematically evaluated the effects of high-pressure processing (HPP, 500 MPa, 10 min) on the comprehensive quality of selenium-sand melon juice (SSJ) over a 35-day refrigerated storage period (4 °C). To simulate extreme contamination scenarios, samples were inoculated with *Escherichia coli* (6.886 log_10_ CFU/mL) and *Aspergillus sclerotioniger* (5.011 log_10_ spores/mL). Five treatment groups were established, including HPP-treated (inoculated and uninoculated) and low-temperature long-time (LTLT, 65 °C for 30 min) pasteurized (inoculated and uninoculated) groups. HPP treatment completely inactivated the inoculated microorganisms, with no regrowth detected during storage, whereas regrowth was observed in the LTLT groups after day 7. HPP-treated samples exhibited superior color stability (ΔE ≤ 1.58) and compounds, and maintained a higher final pH (5.76 vs. 5.39 in LTLT) at the end of storage, with significantly better retention of lycopene and total phenolic compounds compared to LTLT-treated samples. OPLS-DA analysis of volatile compounds showed that the HPP-M group had only four discriminating compounds (VIP ≥ 1), all characteristic C9 aldehydes and alcohols, while the LTLT-M group showed significant acetic acid accumulation, likely due to residual microbial metabolism. This study demonstrates that HPP is superior to LTLT pasteurization for ensuring microbial safety, preserving nutritional quality, and maintaining the characteristic flavor of SSJ during cold storage.

## 1. Introduction

Selenium-sand melon (*Citrullus lanatus*), also known as “*Gobi* Watermelon”, is a characteristic agricultural product of the Ningxia Hui Autonomous Region, China, distinguished by its unique sand-pressing cultivation system and exceptional nutritional profile [1]. This cultivar is particularly enriched in bioactive compounds, including carotenoids (notably lycopene), vitamins, essential amino acids, and trace elements such as selenium, which contribute to its health-promoting properties [2,3]. However, the inherently high moisture content and pronounced heat sensitivity of selenium-sand melon increase its susceptibility to postharvest microbial spoilage and physiochemical deterioration, thereby posing major challenges for storage, transportation, and shelf-life extension [4]. Processing fresh selenium-sand melon into juice represents a viable strategy to reduce postharvest losses while generating value-added products with enhanced economic and commercial potential [5].

Conventional thermal pasteurization techniques, including low-temperature long-time (LTLT, 65 °C for 30 min) and high-temperature short-time (HTST, 85 °C for 15 s) treatments, remain the predominant industrial approaches for ensuring the microbiological safety of fruit juices [6]. Despite their effectiveness in inactivating pathogenic microorganisms, these thermal processes inevitably induce the degradation of heat-labile nutrients, promote adverse color changes, and accelerate the loss of volatile aroma compounds, ultimately compromising both sensory and nutritional quality [7,8]. Lycopene, the predominant carotenoid responsible for the characteristic red hue and potent antioxidant capacity of Selenium-sand melon juice (SSJ), is particularly susceptible to thermal-induced isomerization and oxidative degradation [9,10]. Concurrently, key aroma-active compounds, including aldehydes (such as nonanal and hexanal) and alcohols contributing to fresh and fruity notes, are markedly diminished during thermal processing [11]. These limitations have intensified the search for alternative preservation technologies capable of ensuring microbial safety while maintaining the intrinsic quality attributes of fruit juices.

In recent years, emerging non-thermal processing technologies have gained considerable attention as promising alternatives to conventional thermal treatments for fruit juice preservation [12]. Among these, high-pressure processing (HPP) has been widely used as a commercially viable technology to produce high-quality ready-to-drink juices. HPP involves the application of static pressures ranging from 100 to 1000 MPa, leading to microbial inactivation through mechanisms such as cell membrane disruption, protein denaturation, and enzyme inactivation, while largely preserving low-molecular-weight compounds (vitamins, pigments, and flavor constituents) [13,14]. Numerous studies have demonstrated that HPP-treated juices exhibit superior retention of color, bioactive compounds, antioxidant activity, and sensory characteristics compared with thermally processed counterparts [9]. Recent reviews further highlight the effectiveness of HPP in maintaining antioxidant stability during storage, reinforcing its potential for high-quality fruit product preservation [6]. Additionally, emerging research on watermelon juice processing emphasizes the growing role of innovative technologies, including HPP, in enhancing both safety and quality attributes [15].

Our research group has systematically explored non-thermal processing strategies for Ningxia specialty melon products. In a recent study, Fan et al. [16] employed metagenomic analysis to comprehensively characterize the epiphytic microbial communities on selenium-sand melon surfaces, identifying seven dominant bacterial species and five fungal species as primary contamination sources. Through systematic UV-C inactivation studies and kinetic modeling, they established that the SWeibull2 model provided the most accurate description of fungal inactivation, offering a quantitative framework for process optimization [16]. Extending this work, Liu et al. [17] evaluated the efficacy of induced electric field (IEF) treatment as a non-thermal processing technology for SSJ, demonstrating that IEF2 treatment (60 °C, 88 s) achieved complete microbial inactivation throughout 25 days of refrigerated storage while preserving lycopene, total phenolic content, and characteristic volatile compounds. Furthermore, Li et al. [18] investigated the synergistic effects of UV-C irradiation combined with natural antioxidants (catechin, sodium erythorbate, ascorbic acid, and their combinations) on SSJ, revealing that the catechin–ascorbic acid binary system (HH) exhibited superior preservation of lycopene, antioxidant activity, and flavor stability compared to UV-C alone or thermal pasteurization. These findings emphasize the potential of non-thermal technologies for quality preservation of melon-based products, yet the application of HPP as a standalone technology for SSJ under extreme contamination conditions remains unexplored.

Unlike UV-C irradiation, which is primarily limited by juice turbidity and optical penetration depth, HPP offers the distinct advantage of uniform pressure transmission independent of product optical properties, making it particularly suitable for opaque and turbid juices such as selenium-sand melon juice [19]. Moreover, while our earlier work [18] demonstrated the effectiveness of antioxidant combinations in mitigating oxidative degradation during UV-C treatment, the present study focuses on HPP as a standalone technology to systematically evaluate its intrinsic capacity for microbial inactivation and quality preservation without the confounding effects of added preservatives. HPP has been shown to inactivate microorganisms through multiple mechanisms, including cell membrane disruption, protein denaturation, and enzyme inactivation, which collectively contribute to its superior sustained antimicrobial effect compared to thermal treatments [20,21].

Although previous studies have investigated the effects of HPP on watermelon juice, most have focused on selected quality attributes (e.g., microbial inactivation, color, or antioxidant activity) over relatively short storage periods, and none have combined a 35-day multi-parameter quality assessment with deliberate contamination challenge testing and chemometric flavor profiling within a single experimental design [6,9,15]. Furthermore, the efficacy of HPP under extreme contamination scenarios—a critical consideration for industrial application where raw materials may carry high initial microbial loads—remains insufficiently explored. The systematic evaluation of HPP technology using both uninoculated and deliberately contaminated samples provides a more rigorous and practically relevant assessment of its processing capability and safety margins. Additionally, the comprehensive characterization of volatile flavor compounds using advanced chemometric approaches, such as orthogonal partial least squares discriminant analysis (OPLS-DA), offers unprecedented insights into the underlying quality retention during storage [17,18]. Recent meta-analyses have highlighted that hurdle approaches combining non-thermal technologies with other preservation factors can achieve synergistic effects, yet the specific application of HPP as a standalone technology under high-contamination conditions warrants systematic investigation [19].

The primary objective of this study was therefore to systematically evaluate the effects of HPP treatment (500 MPa, 10 min) on the comprehensive quality of SSJ during 35 days of refrigerated storage at 4 °C, using both uninoculated and inoculated samples containing *Escherichia coli* D25015 and *Aspergillus sclerotioniger* to simulate extreme contamination conditions. Conventional LTLT pasteurization (65 °C, 30 min) served as the benchmark control. Quality parameters assessed included microbial stability, color attributes, pH, soluble solids, titratable acidity, lycopene content, total phenolics, antioxidant activity, and volatile flavor compound profiles analyzed by HS-SPME-GC-MS coupled with multivariate statistical analysis. The study provides a theoretical and experimental foundation supporting the industrial application of HPP to produce high-quality, safe, and shelf-stable SSJ, while addressing critical knowledge gaps regarding its performance under challenging processing conditions and complementing our previous research on non-thermal processing of Ningxia specialty melons [16,17,18].

## 2. Materials and Methods

### 2.1. Materials

The experimental raw material, selenium-sand melon (*Citrullus lanatus*) of the variety *Jinhua No. 6* was purchased from a local market in Ningxia Hui Autonomous Region, China. Disease-free, undamaged fruits of uniform size and ripeness were carefully selected for juice preparation.

#### 2.1.1. Strain Cultivation and Preparation of Microbial Suspensions

For the preparation and inoculation of fungal suspension, a sample of *A. sclerotioniger* glycerol lyophilizate (a strain preserved in this laboratory) was picked up using an inoculation loop and purified by the three-point streaking method on Potato Dextrose Agar (PDA) plates (Haibo Biotechnology Co., Ltd., Qingdao, China). The plates were incubated at 28 °C for 72 h. Fresh spores were collected from the edge of a single colony and transferred via single-point inoculation to PDA slant medium. The inoculated test tubes were incubated at 28 °C for 72 h to obtain well-grown fungal mycelium and spores. Subsequently, 7.5 mL of sterile phosphate-buffered saline (PBS) buffer (Beijing Lanjieke Technology Co., Ltd., Beijing, China) was added to the slant test tube in two separate additions. The spores were gently scraped with an inoculation loop to disperse them in the buffer, and the mixture was filtered through a 70 μm sterile cell filter to remove mycelial fragments. The collected filtrate constituted the *A. sclerotioniger* spore suspension. The spore concentration was adjusted to approximately 10^7^ spores/mL using a hemocytometer. The spore suspension was inoculated into the SSJ sample at a 1% (*v*/*v*) inoculation rate to achieve a final concentration of approximately 10^5^ spores/mL, which served as the target microorganism for subsequent evaluation of microbial inactivation efficacy [16].

For the preparation and inoculation of bacterial suspension, *E. coli D25015* (Shanghai Bio-Resource Center, Shanghai, China) was cultured in Brain Heart Infusion (BHI) broth at 37 °C for 12 h to reach the late logarithmic growth phase. Thirty milliliters of the bacterial culture was transferred to a 50 mL sterile centrifuge tube and centrifuged at 4 °C and 10,000 rpm for 1 min. The supernatant was discarded, and the bacterial pellet was resuspended in sterile PBS buffer. The washing procedure was repeated four times. After the final wash, the bacterial pellet was resuspended in a small volume of PBS, and the concentration was adjusted to approximately 10^9^ CFU/mL using the plate count method [16]. The target organism for the subsequent assessment of microbial inactivation efficacy was the prepared *E. coli* suspension, which was inserted into the SSJ sample at a 1% (*v*/*v*) inoculation rate to generate a final concentration of about 10^7^ CFU/mL [16].

#### 2.1.2. Juicing and HPP Treatment

Disease-free, undamaged selenium-sand melons (‘*Jinhua No. 6*’) of uniform size and ripeness were selected, washed thoroughly with distilled water, cut into wedges, peeled, and seeded. The flesh was then cut into small, uniform pieces. Juice was extracted using a commercial juicer (SJ-01, Mengda, Ningbo, China). The extracted juice was filtered through an 80-mesh strainer to separate it from the pulp and remove coarse particles. The freshly prepared selenium-sand melon juice (SSJ) was immediately filled into 30 mL sterile high-density polyethylene (HDPE) wide-mouth reagent bottles and refrigerated at 4 °C until further use. All inoculated samples were treated within 30 min after inoculation, and uninoculated controls were processed in parallel under identical conditions.

**Figure 1:**
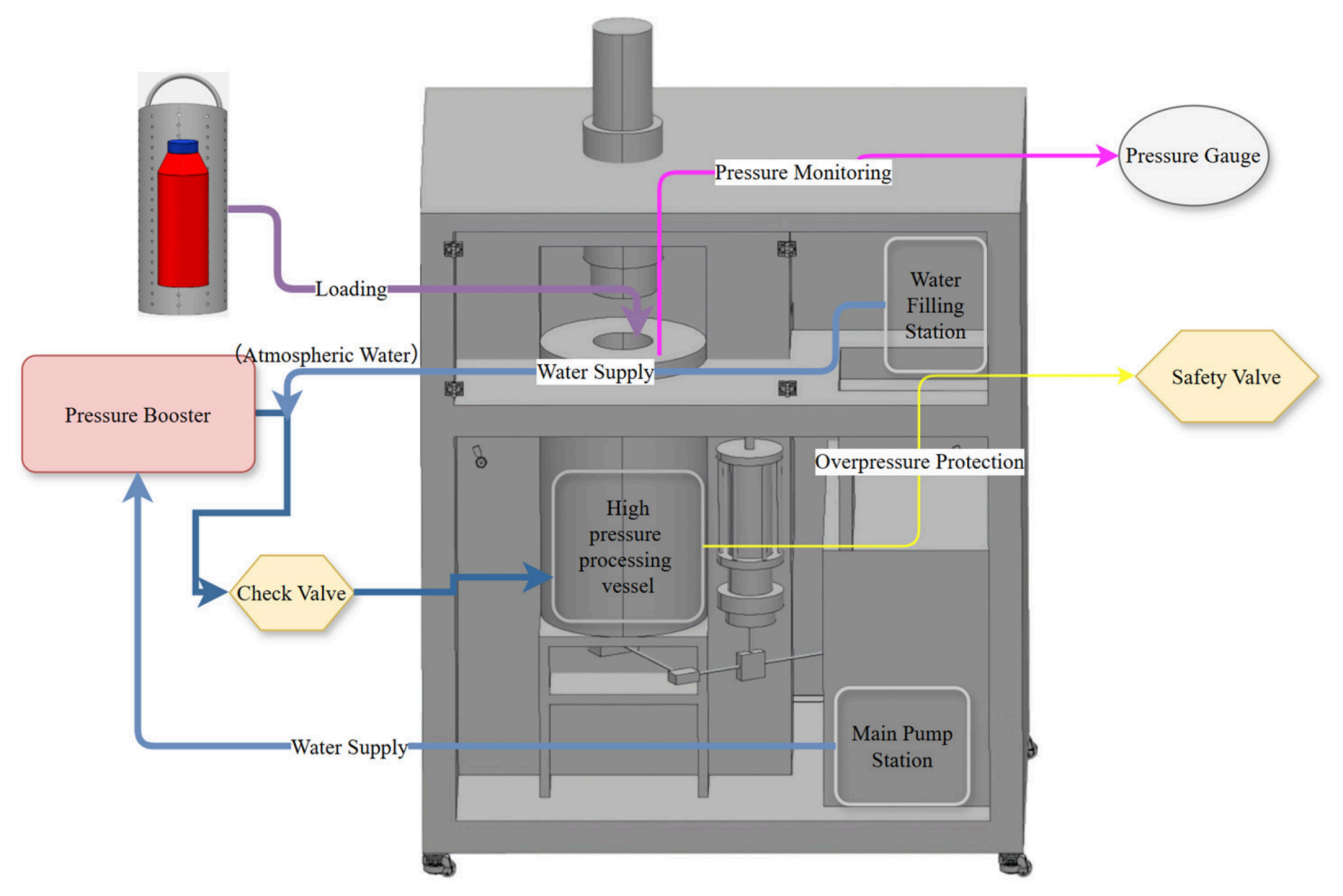
Schematic diagram of the HPP apparatus.

HPP treatment was performed using a 5 L HPP sterilization system (BY600-5L, Wenzhou Bin Yi Machinery Technology Co., Ltd., Wenzhou, China). This system (Figure 1) uses a hydraulically driven pressurizer to pump high-pressure water into a circular high-pressure chamber (inner diameter 120 mm, length 550 mm), generating a hydrostatic pressure of up to 600 MPa. Samples were loaded into the chamber using porous metal baskets, with water serving as the pressure-transmitting medium. The chamber was sealed by a hydraulically driven gate-type frame and end caps. The pressurizer raised the internal pressure to the set value and maintained it for the predetermined time, after which the pressure was automatically released via a relief valve.

SSJ samples in sterile HDPE wide-mouth reagent bottles were placed inside the treatment chamber. Using water as the pressure-transmitting medium, the samples were subjected to a pressure of 500 MPa for 10 min at room temperature (approximately 25 °C). The pressure come-up time was approximately 3 min, and the pressure release was instantaneous. Three independent replicate treatments were conducted for each sample group. Pasteurization was performed in a water bath (HH-S4, Changzhou Guoyu Instrument Manufacturing Co., Ltd., Changzhou, China) under the following conditions: 65 °C for 30 min (LTLT). After treatment, all samples were immediately placed in an ice-water bath and rapidly cooled to room temperature. All treated samples were stored at 4 °C for 35 days, and analyses were conducted on days 0, 7, 14, 21, 28, and 35.

#### 2.1.3. Experimental Groups

The experiment included five treatment groups: freshly squeezed SSJ control group (CK group) with no treatment; HPP-treated uninoculated group (HPP group, 500 MPa, 10 min); HPP-treated group with added microorganisms (HPP-M group, 500 MPa, 10 min); LTLT pasteurized uninoculated group (LTLT group, 65 °C, 30 min); and LTLT pasteurized group with added microorganisms (LTLT-M group, 65 °C, 30 min). The 30 min holding time was counted from the moment the samples were placed into the water bath, not from the moment the juice temperature reached 65 °C. The LTLT treatment was performed in a digital thermostatic water bath (HH-S4, Changzhou Guoyu Instrument Manufacturing Co., Ltd., Changzhou, China). Because the samples were processed in sealed containers, the juice temperature inside the bottles could not be directly measured; therefore, the water bath temperature was monitored via its digital temperature display and maintained at 65 ± 0.5 °C throughout the hold. Considering the small fill volume (30 mL) and the direct immersion in the preheated bath, the come-up time of the juice was expected to be short. All samples were stored at 4 °C for 35 days, with analyses performed at 7-day intervals.

### 2.2. Microbial Counting

For microbial enumeration, SSJ samples were serially diluted with sterile PBS buffer and spread onto the appropriate culture media: BHI agar (Haibo Biotechnology Co., Ltd., Qingdao, China) for *E. coli enumeration*, PDA for *A. sclerotioniger* enumeration, and Plate Count Agar (PCA) (Haibo Biotechnology Co., Ltd., Qingdao, China) for total aerobic bacterial counts in uninoculated control groups. BHI agar plates were incubated at 37 °C for 24 h, PDA plates at 28 °C for 48 h, and PCA plates at 37 °C for 48 h. After incubation, colonies were counted, and the results were expressed as log_10_ CFU mL^−1^. Three replicate plate counts were performed for each sample, and the mean values were calculated [18]. The detection limit was 1 log_10_ CFU mL^−1^.

### 2.3. Color Measurement

Color parameters were measured using a DS-400 spectrophotometer (CaiPu Technology Zhejiang Co., Ltd., Hangzhou, China) at 25 °C in reflectance mode with D/8° geometry, using the D65 illuminant, the 10° standard observer, the specular-component-included (SCI) mode, and an 11 mm aperture (400–700 nm at 10 nm intervals), with the instrument calibrated against a standard white plate before measurement. After thorough mixing, 30 mL of SSJ sample was transferred into a quartz cuvette, and the CIELAB color parameters L* (lightness), a* (red-green value), and b* (yellow-blue value) were recorded. The total color difference (ΔE) was calculated using the following formula [22]:

$$\Delta E=\sqrt{{\left(L_{n}^{*}-L_{m}^{*}\right)}^{2}+{\left(a_{n}^{*}-a_{m}^{*}\right)}^{2}+{\left(b_{n}^{*}-b_{m}^{*}\right)}^{2}}$$

L* represents the sample’s lightness, a* represents the red-green value, and b* represents the yellow-blue value; the subscripts m and n represent the measured values of the initial sample and the treated sample, respectively.

### 2.4. Determination of Total Soluble Solids and Titratable Acids

Total soluble solids (TSS) content was determined using a handheld refractometer with automatic temperature compensation (ATC) (Chengdu Optical Accessories Factory, Chengdu, China). Prior to measurement, the refractometer was calibrated with distilled water. Subsequently, 0.2 mL of the mixed SSJ sample was dispensed using a disposable pipette tip, evenly spread onto the measuring prism, and the cover plate was closed. The reading was recorded under natural light, and results were expressed in °Brix. Each sample was tested in triplicate, and the average value was calculated.

Titratable acidity (TA) was determined by acid–base titration following the method of Liu et al. [23]. Five milliliters of SSJ were transferred to a 100 mL volumetric flask and diluted to volume with distilled water. The diluted solution was transferred to a 250 mL conical flask, and 3 drops of phenolphthalein indicator (1% *w*/*v* in ethanol) were added. The solution was titrated with 0.05 mol/L NaOH standard solution until the solution turned pale red and did not fade within 30 s. The TA content was expressed as grams of citric acid equivalent per 100 mL of juice (g CAE/100 mL), calculated using the following formula [23]:

$$TA=100\times \frac{V_{1}\times C\times 0.064}{V_{2}}$$

where V_1_ is the volume of NaOH standard solution consumed (mL); C is the molar concentration of the NaOH standard solution (0.05 mol/L); V_2_ is the sample volume (mL); 0.064 is the conversion factor based on citric acid.

### 2.5. pH Determination

The pH of SSJ was measured using a digital pH meter (Seven Compact S210, Mettler Toledo, Greifensee, Switzerland) equipped with a combined glass electrode. The pH meter was calibrated with standard buffer solutions (pH 4.01, 7.00, and 10.01) prior to measurement. After thorough mixing, the sample was equilibrated to room temperature (25 ± 1 °C), and the pH reading was recorded. Each sample was measured in triplicate, and the mean value was calculated [24].

### 2.6. Methods for Determining Total Sugars and Reducing Sugars

Total sugars and reducing sugars were determined using the 3,5-dinitrosalicylic acid (DNS) colorimetric method following the procedure of Liu et al. [17]. For total sugar determination, 1 mL of SSJ was mixed with 15 mL distilled water and 10 mL of 6 M HCl, then hydrolyzed in a boiling water bath for 30 min. After cooling, the solution was neutralized with 6 M NaOH using phenolphthalein as indicator, diluted to 100 mL, and filtered. One milliliter of the diluted solution was mixed with 1 mL of DNS reagent, heated in a boiling water bath for 5 min, cooled, and diluted to 10 mL with distilled water. Absorbance was measured at 540 nm using a spectrophotometer. For reducing sugar determination, the same procedure was followed without the acid hydrolysis step. A calibration curve was established using a glucose standard solution, and the resulting regression equation was y = 0.3879x − 0.0187 (R^2^ = 0.998).

### 2.7. Determination of Lycopene Content

Lycopene content was determined spectrophotometrically following the method of Sevindik Baç et al. [25]. A mixture of n-hexane, acetone, and anhydrous ethanol in a volume ratio of 2:1:1 (*v*/*v*/*v*) was used as the lycopene extraction solvent. One milliliter of SSJ was mixed with 25 mL of the extraction solvent and magnetically stirred at 4 °C for 15 min in the dark to prevent photodegradation. Subsequently, 15 mL of distilled water was added, and the mixture was stirred for an additional 5 min. The mixture was allowed to stand for 15 min to allow phase separation. The upper n-hexane layer (containing extracted lycopene) was carefully aspirated, and its absorbance was measured at 503 nm using a spectrophotometer. Quantification was performed using a lycopene standard curve (y = 0.1471x + 0.0064, R^2^ = 0.997), and results were expressed as μg lycopene per mL of juice (μg/mL). All extractions were performed in triplicate under dim light to minimize lycopene degradation.

### 2.8. Method for Determining Total Phenolic Content

Total phenolic content (TPC) was determined using the Folin–Ciocalteu colorimetric method following the procedure of Pinto et al. [26]. One milliliter of SSJ was transferred to a 10 mL volumetric flask and diluted to volume with distilled water. One milliliter of the diluted solution was transferred to a test tube, followed by the addition of 1 mL of Folin–Ciocalteu reagent (diluted 1:10 with distilled water). The mixture was vortexed and allowed to stand in the dark for 5 min. Subsequently, 0.8 mL of 200 g/L Na_2_CO_3_ solution was added, and the volume was adjusted to 10 mL with distilled water. The mixture was incubated at 30 °C for 30 min in the dark, and the absorbance was measured at 765 nm using a spectrophotometer. A calibration curve was constructed using gallic acid standards (0–200 μg/mL), and results were expressed as milligrams of gallic acid equivalents per 100 mL of juice (mg GAE/100 mL). Each sample was analyzed in triplicate.

### 2.9. Determination of DPPH Radical Scavenging Rate

DPPH radical scavenging activity was determined according to the method of Ni et al. [27]. One milliliter of SSJ was mixed with 1 mL of 0.2 mmol/L DPPH ethanolic solution in a test tube. The mixture was vortexed and incubated at room temperature (25 °C) in the dark for 30 min. The absorbance was measured at 517 nm using a spectrophotometer. A blank control was prepared by replacing the sample with 1 mL of anhydrous ethanol, and a sample blank was prepared by replacing the DPPH solution with 1 mL of anhydrous ethanol. The DPPH radical scavenging activity was calculated using the following formula [27]:

$$DPPH\;Radical\;Scavenging\;Rate\;(\%)=[1-(Ai-Aj)/A_{0}]\times 100\%$$

where Ai is the absorbance after the reaction of SSJ with the DPPH solution; Aj is the absorbance after the reaction of SSJ with anhydrous ethanol; A_0_ is the absorbance after the reaction of anhydrous ethanol with the DPPH solution.

### 2.10. Determination of ABTS Radical Scavenging Rate

ABTS radical scavenging activity was determined using a commercial ABTS assay kit (Suzhou Keming Biotechnology Co., Ltd., Suzhou, China) following the manufacturer’s instructions with modifications based on the method of Błaszczak et al. [28]. The ABTS^+^ radical cation was generated by reacting 7 mmol/L ABTS stock solution with 2.45 mmol/L potassium persulfate (1:1, *v*/*v*) and incubating in the dark at room temperature for 16 h. The ABTS^+^ working solution was diluted with ethanol to an absorbance of 0.700 ± 0.020 at 734 nm. Twenty microliters of SSJ were mixed with 980 μL of the diluted ABTS^+^ working solution and incubated in the dark at room temperature for 6 min. The absorbance was measured at 734 nm using a spectrophotometer. Results were expressed as μmol Trolox equivalents per mL of juice (μmol TE/mL) using a Trolox standard curve. All measurements were performed in triplicate.

### 2.11. SPME and GC-MS

Volatile organic compounds (VOCs) were extracted and analyzed using headspace solid-phase microextraction coupled with gas chromatography–mass spectrometry (HS-SPME-GC-MS). A GC-MS system (GCMS-TQ8040 NX, Shimadzu Corporation, Kyoto, Japan) equipped with a PAL3 autosampler was employed for the analysis.

Headspace Solid-Phase Microextraction (HS-SPME): Eight milliliters of SSJ were transferred to a 20 mL headspace vial containing 3 g of NaCl, and 20 μL of internal standard solution (1,2-dichlorobenzene, 20 μg/mL in methanol) was added. The vial was immediately sealed with a PTFE/silicone septum and placed in the PAL3 autosampler. The sample was equilibrated at 40 °C for 20 min with agitation (250 rpm). A 50/30 μm DVB/CAR/PDMS SPME fiber (Supelco, Bellefonte, PA, USA) was pre-conditioned at 250 °C for 10 min in the GC injection port. The fiber was then inserted through the septum into the headspace vial, and VOCs were extracted at 40 °C for 20 min with continuous agitation. After extraction, the fiber was withdrawn and immediately transferred to the GC injection port, where thermal desorption was performed at 250 °C for 5 min in splitless mode. After each desorption, the fiber was conditioned at 250 °C for 3 min to remove residual compounds.

GC Conditions: Helium (purity ≥ 99.999%) was used as the carrier gas under constant-pressure control with a pre-column pressure of 100.0 kPa, a linear velocity of 48.1 cm/s, and a corresponding column flow rate of 1.78 mL/min. The injector temperature was maintained at 250 °C. Injection was performed in splitless mode with a splitless time of 1.00 min, and the purge flow rate was 3.0 mL/min. Volatile compounds were separated using a DB-5MS capillary column (30 m × 0.25 mm i.d., 0.25 μm film thickness; Agilent Technologies, Santa Clara, CA, USA). The column temperature program was as follows: initial temperature 40 °C held for 2 min; ramped at 4 °C/min to 140 °C and held for 3 min; then ramped at 8 °C/min to 220 °C and held for 5 min. The total run time was 44 min.

MS Conditions: The mass spectrometer was operated in electron impact (EI) ionization mode at 70 eV. The ion source temperature was set at 230 °C, and the GC-MS interface temperature was maintained at 250 °C. A solvent delay of 1.0 min was applied to protect the filament. Mass spectra were acquired in full-scan mode with a scan range of m/z 40–500, an event cycle of 0.3 s, and a scan rate of 1666 amu/s. Compound identification was performed by comparing the mass spectra with the NIST 20 spectral library (National Institute of Standards and Technology, Gaithersburg, MD, USA); results were accepted only when the similarity match was ≥80%. For compounds not available in the library, identification was based on retention indices calculated using a series of n-alkanes (C_8_–C_20_) and compared with literature values. Quantification was performed using the internal standard method with 1,2-dichlorobenzene as the internal standard, and concentrations were expressed as μg/mL of juice equivalent. LOD (S/N = 3) ≈ 0.02 μg/mL and LOQ (S/N = 10) ≈ 0.08 μg/mL, estimated from replicate peak signal-to-noise data across all samples. The “0.0000” entries in Table S1 represent compounds without an integrated peak (below the LOD) and have been replaced with “ND.” (not detected); the abrupt drops and recoveries therefore reflect time points where concentrations fluctuated around the LOD rather than genuine disappearance/reappearance of the compounds.

### 2.12. Statistical Analysis

All experiments were conducted in triplicate using independently prepared samples, and results are expressed as mean ± standard deviation (SD). Both one-way and two-way ANOVA were performed using IBM SPSS Statistics 24 software (IBM Corp., Armonk, NY, USA), and Duncan’s multiple range test was used to assess the significance of differences between treatment groups at a significance level of *p* < 0.05. For volatile compound analysis, multivariate statistical analyses including principal component analysis (PCA) and orthogonal partial least squares discriminant analysis (OPLS-DA) were performed using SIMCA 14.1 software (Umetrics, Umeå, Sweden).

## 3. Results and Discussion

### 3.1. Effects of Different Treatments on the Microbial Safety of SSJ During Storage

**Figure 2:**
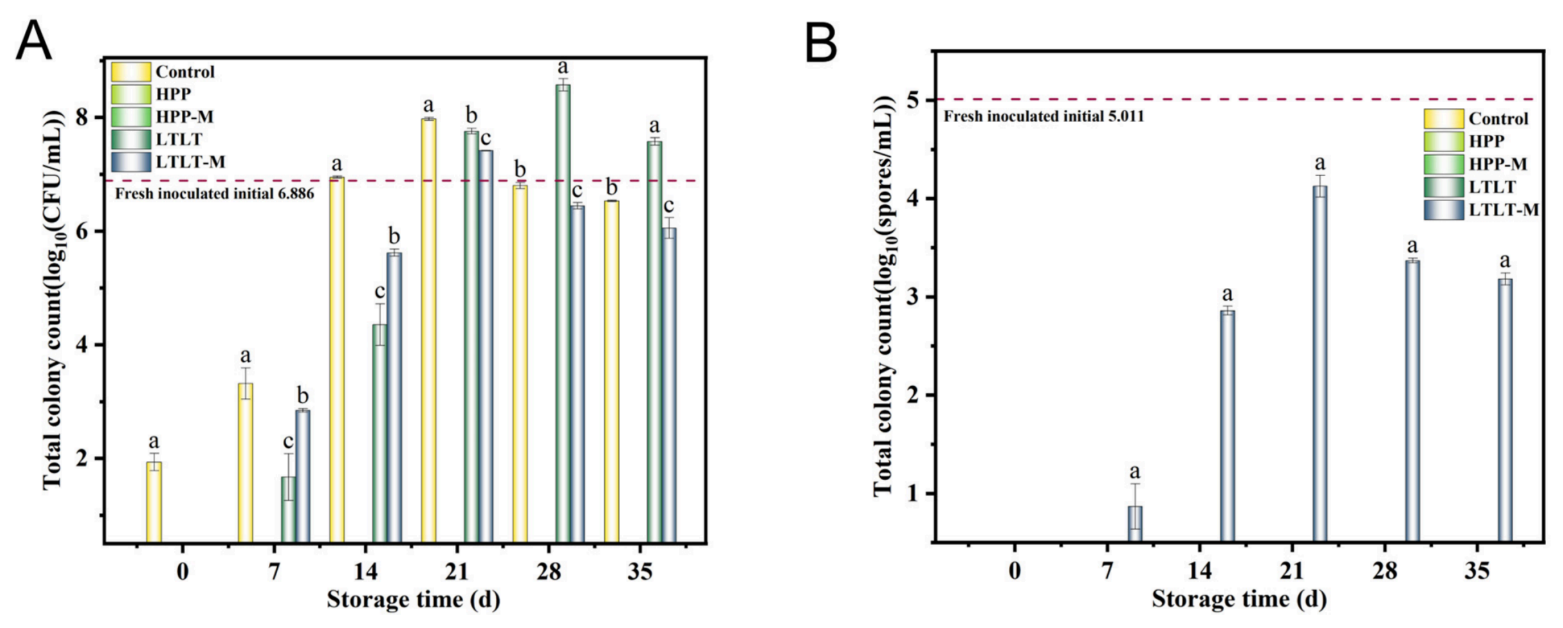
Changes in total colony counts of *E. coli* (**A**) and *A. sclerotioniger* (**B**) in different treated SSJ samples for 35-day storage. Values are presented as mean ± SD (n = 3). Different letters at the same storage time indicate significant differences among treatments (Duncan’s multiple range test, *p* < 0.05).

The initial microbial loads of inoculated SSJ samples were 6.886 log_10_ CFU mL^−1^ for *E. coli* and 5.011 log_10_ spores mL^−1^ for *A. sclerotioniger* (Figure 2A,B). HPP treatment at 500 MPa for 10 min effectively reduced both pathogens to undetectable levels, and no colonies were detected in the uninoculated HPP group throughout the 35-day storage period. In contrast, while LTLT treatment (65 °C, 30 min) initially eliminated detectable pathogens in the LTLT-M group, regrowth was observed after 7 days of refrigerated storage, with *E. coli* counts rebounding to 2.114 log_10_ CFU mL^−1^ (LTLT) and 2.875 log_10_ CFU mL^−1^ (LTLT-M), and *A. sclerotioniger* rebounding to 0.867 log_10_ spores mL^−1^ (LTLT-M), exceeding initial inoculation levels. The untreated control (CK) group exhibited rapid microbial proliferation, reaching 7.976 log_10_ CFU mL^−1^ by day 21. HPP irreversibly disrupts microbial cell membranes, impairing permeability and ion-exchange functions, while also denaturing replication-essential proteins and inactivating key enzymes, thereby blocking the repair pathways available to sublethally injured cells [20,29]. These findings align with our earlier UV-C inactivation study on SSJ, where our model best described the nonlinear inactivation kinetics of fungal spores, reflecting heterogeneous stress sensitivity within microbial populations [16]. Notably, HPP outperforms UV-C in turbid matrices, as UV-C efficacy is limited by optical penetration depth, a constraint absent in HPP’s pressure-based mechanism [16,17,18,21]. The regrowth observed in LTLT-treated samples indicates that sub lethally injured cells can repair and resume proliferation during refrigerated storage [30], consistent with reports of heat-resistant or sub lethally damaged cells recovering in pasteurized juices [31]. By comparison, IEF2 treatment (60 °C, 88 s) in our earlier selenium-sand melon juice study achieved complete sterilization for 25 days, comparable to HPP’s efficacy here [17]. However, HPP offers uniform pressure transmission independent of sample conductivity, a prerequisite for IEF efficacy [17,19]. The absence of regrowth in HPP-treated samples over 35 days confirms its predominantly lethal (rather than sublethal) mechanism of action, supporting its reliability as a non-thermal sterilization technology for selenium-sand melon juice.

### 3.2. Effects of Different Treatments on the Color of SSJ During Storage

**Figure 3:**
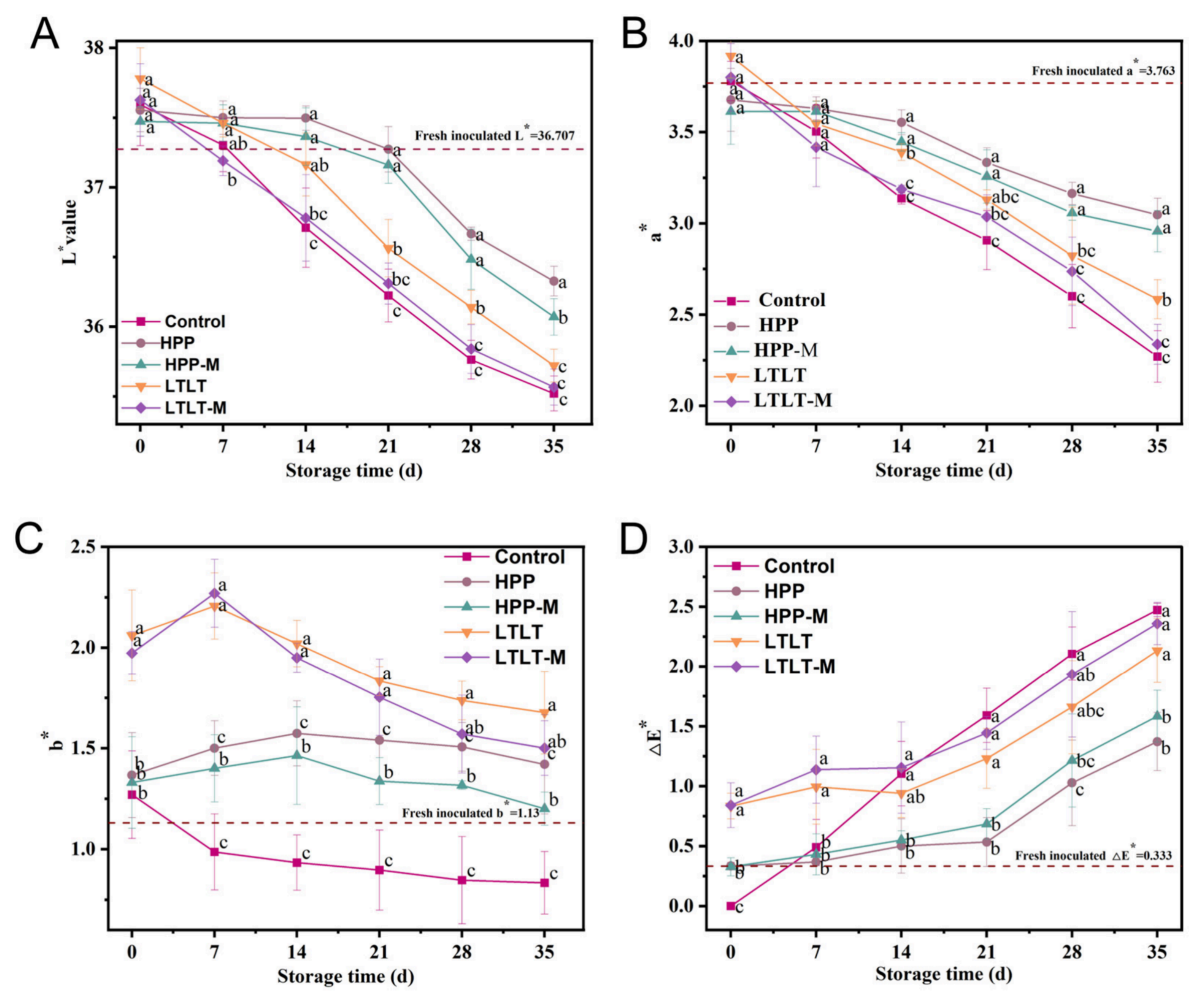
Changes in color parameters of SSJ subjected to various treatments throughout 35-day storage: (**A**) L*, (**B**) a*, (**C**) b*, (**D**) ΔE. Values are presented as mean ± SD (n = 3). Different letters at the same storage time indicate significant differences among treatments (Duncan’s multiple range test, *p* < 0.05).

Color parameters (L, a, b, ΔE) of SSJ during 35 days of storage at 4 °C are shown in Figure 3. L values (lightness) declined across all groups (Figure 3A), but significantly less in HPP-treated samples—decreasing only 1.23 units (HPP, to 36.33) and 1.40 units (HPP-M, to 36.07)—compared to CK, LTLT, and LTLT-M. This decline reflects combined enzymatic browning (via PPO and POD) and non-enzymatic reactions, including ascorbic acid degradation, Maillard reactions, and phenolic oxidation [21]. These results align with our prior findings on IEF-treated selenium-sand melon juice, where non-thermal processing minimized enzymatic browning by inactivating PPO while avoiding heat-accelerated non-enzymatic browning [17].

The a* values (redness) declined in all groups (Figure 3B), reflecting lycopene degradation, watermelon’s primary red pigment [23]. HPP and HPP-M showed significantly smaller decreases, maintaining higher redness throughout storage. Although LTLT initially exhibited slightly higher a* values, it declined substantially during storage; CK showed the greatest redness loss. Superior a* retention under HPP correlates with lycopene preservation, as HPP limits oxidative degradation and isomerization of carotenoids [9,10]. This is consistent with our UV-C/antioxidant study on selenium-sand melon juice, where a catechin–ascorbic acid system reduced lycopene cis-isomerization by 72% relative to UV-C alone [18]. Notably, HPP alone achieves comparable lycopene protection without exogenous antioxidants, likely through inactivation of lipoxygenase and other oxidative enzymes involved in carotenoid degradation [7,21].

The b values (yellow-blue hue) showed minimal fluctuation in HPP-treated samples, indicating superior color stability (Figure 3C). Total color difference (ΔE) remained below 2 for HPP (max 1.37) and HPP-M (max 1.58) throughout storage, while CK, LTLT, and LTLT-M exceeded this threshold, indicating visually perceptible color changes (Figure 3D) [22], since ΔE ≤ 2 is generally considered imperceptible to the human eye [22].

HPP’s color-preserving effect primarily results from minimal disruption of pigment covalent bonds, preventing high-temperature-induced non-enzymatic browning and pigment isomerization [7], along with effective inactivation of PPO and POD [21]. These results align with Liu et al.’s findings on IEF-treated selenium-sand melon juice, where IEF similarly mitigated color deterioration during storage [17]. Notably, HPP demonstrated superior color stability over a longer storage period (35 vs. 25 days), suggesting that substantial (albeit not necessarily complete) inactivation of oxidative enzymes under high pressure provides more durable protection against color degradation; residual PPO/POD activity after HPP has been reported at similar pressure levels [14,21], which is consistent with the slight L* decline observed in the HPP groups.

### 3.3. Effects of Different Treatments on Key Parameters of SSJ During Storage

**Figure 4:**
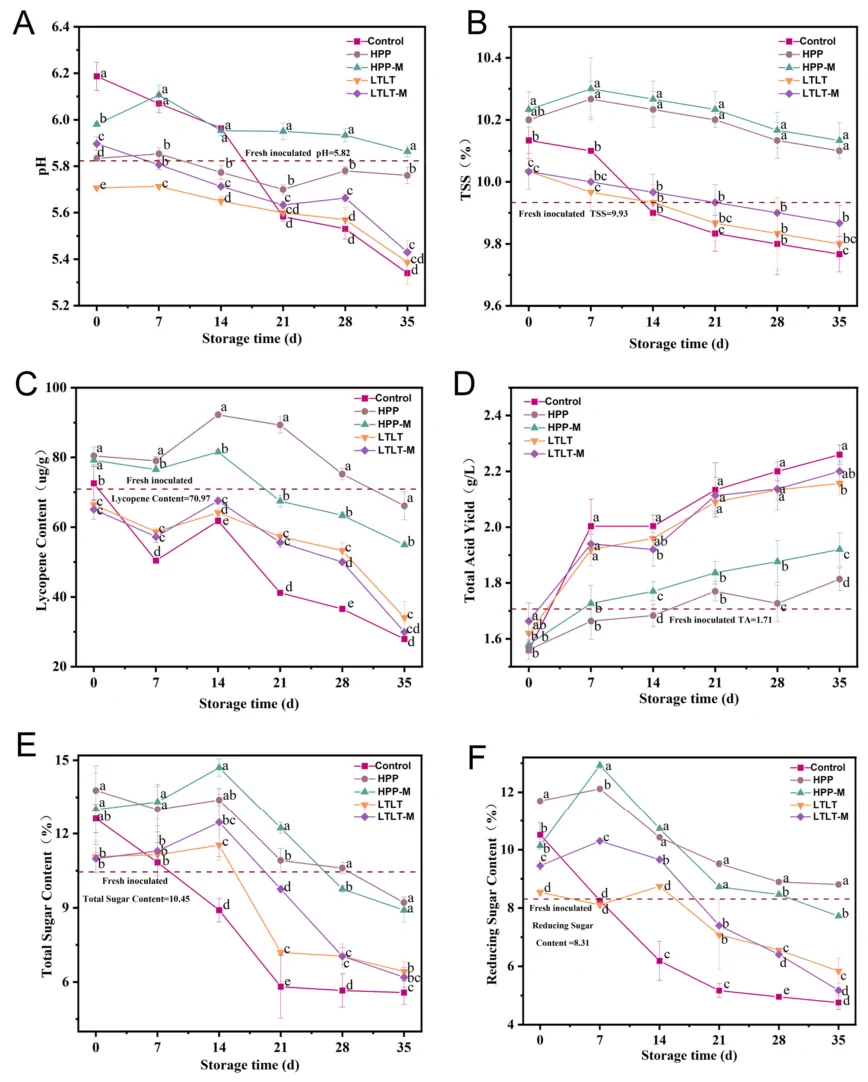
Changes in physicochemical properties of SSJ subjected to various treatments over 35-day storage period: (**A**) pH, (**B**) TSS, (**C**) lycopene, (**D**) titratable acid, (**E**) total sugars, (**F**) reducing sugars. Values are presented as mean ± SD (n = 3). Different letters at the same storage time indicate significant differences among treatments (Duncan’s multiple range test, *p* < 0.05).

The pH of all groups declined during storage (Figure 4A), reflecting microbial metabolic activity and organic acid accumulation. The HPP group showed the smallest decrease and greatest pH stability, consistent with Aganovic et al. [32], who reported that HPP inactivates microorganisms via membrane damage and protein denaturation, preventing metabolic acid production. In contrast, LTLT and LTLT-M groups exhibited continuous decline that accelerated after day 21, reaching pH 5.39 and 5.43, respectively, by day 35—significantly lower than the HPP group (5.76)—indicating incomplete microbial inactivation and subsequent acid-producing proliferation [33]. This parallels our earlier UV-C study, where only UV-C combined with sodium erythorbate achieved complete bacteriostasis and pH stability [18].

Total soluble solids (TSS) increased following HPP treatment relative to CK, whereas LTLT decreased TSS (Figure 4B), consistent with Zhu et al. [34] and our IEF study [17]. This likely reflects pressure-induced cell wall disruption releasing bound soluble solids [7].

Lycopene content declined overall across all groups despite fluctuations (Figure 4C), with HPP showing the least loss, followed by HPP-M; CK and LTLT showed substantial degradation. This decline reflects oxidative stress and microbial activity accelerating carotenoid breakdown [23]. HPP preserved lycopene by inactivating microorganisms and oxidative enzymes while minimally affecting small-molecule compounds [7]—achieving retention comparable to antioxidant-combined UV-C treatments in our earlier study [18], despite requiring no exogenous additives.

Titratable acidity (TA) increased across all groups (Figure 4D), reflecting acid accumulation; HPP significantly limited this increase through microbial and enzymatic inactivation. Total and reducing sugars declined similarly (Figure 4E,F), with HPP showing the highest retention, significantly exceeding CK. This sugar loss is primarily attributable to microbial metabolism and residual enzymatic activity, consistent with our IEF findings, where treated samples maintained more stable sugar profiles than untreated controls [17].

Overall, HPP outperformed LTLT across all physicochemical parameters—pH stability, TSS retention, lycopene preservation, TA control, and sugar protection—by simultaneously inactivating microorganisms and endogenous enzymes, suppressing quality deterioration at its source. Notably, HPP maintained quality even under high contamination (HPP-M), confirming its reliability for selenium-sand melon juice processing. These results position HPP as a robust option for opaque juices, where optical (UV-C) and conductivity-dependent (IEF) technologies face inherent constraints [16,17,18].

### 3.4. Effects of Different Treatments on the Antioxidant Activity of SSJ During Storage

**Figure 5:**
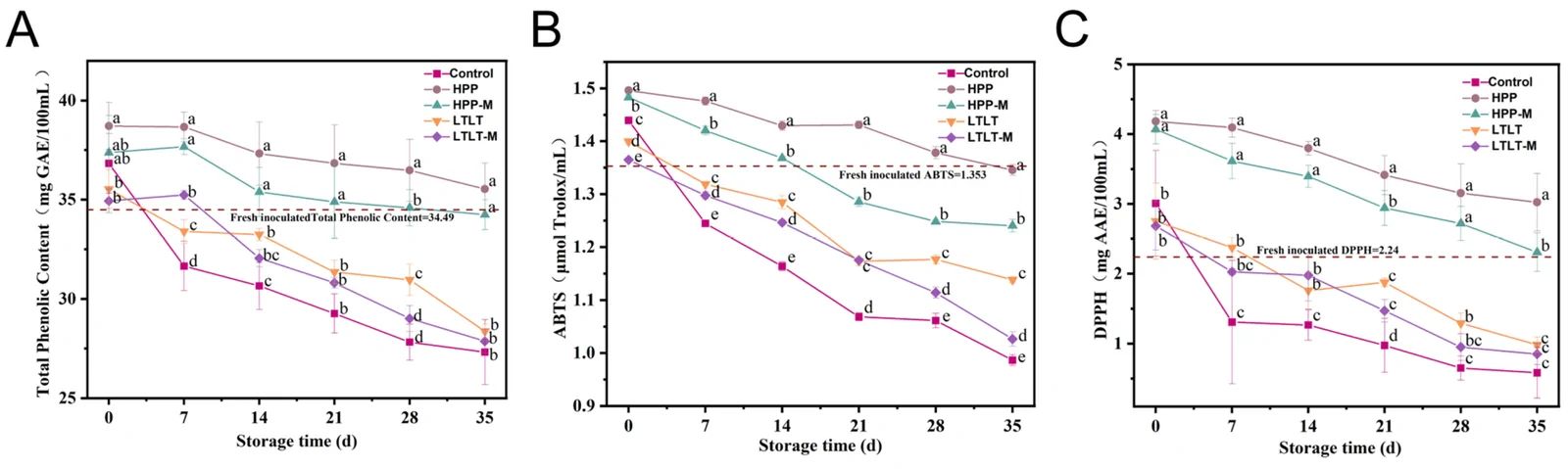
Changes in antioxidant capacity and total phenolic content of SSJ subjected to various treatments during 35-day storage: (**A**) total phenolics, (**B**) ABTS radical scavenging activity, (**C**) DPPH radical scavenging activity. Values are presented as mean ± SD (n = 3). Different letters at the same storage time indicate significant differences among treatments (Duncan’s multiple range test, *p* < 0.05).

Antioxidant activity of SSJ declined across all treatment groups during 35 days of storage at 4 °C, though decline rates differed significantly (Figure 5). Total phenolic content (TPC) decreased continuously, with HPP showing the highest retention, followed by LTLT, and CK the lowest (Figure 5A). The superior pH retention in HPP-treated samples is consistent with findings by Aganovic et al. [32] but diverges from the observations of Liu et al. [23], who reported that HPP-treated watermelon juice still exhibited a gradual pH decline over 28 days, albeit slower than thermal controls. Variations in the initial microbial load, storage temperature, or the watermelon cultivar utilized could be the cause of this disparity. Notably, the sublethal harm and subsequent recovery reported in other HPP investigations under less strict conditions contrast with the total inactivation of inoculated *E. coli* and *A. sclerotioniger* in our HPP groups, with no regrowth throughout 35 days [30,31]. In these test organisms, the 500 MPa/10 min treatment seems to surpass the threshold for irreparable harm, offering a more robust safety margin than the 400–450 MPa treatments often reported in the literature for fruit juices [20,21]. These results parallel our UV-C study, where the catechin–ascorbic acid system (HH) retained 92% of initial TPC after 4 weeks [18]. Notably, HPP alone achieved comparable retention without exogenous antioxidants, reflecting its intrinsic effectiveness in enzyme inactivation and oxidative protection.

ABTS and DPPH radical scavenging activities followed trends consistent with TPC (Figure 5B,C), confirming phenolic compounds as the primary antioxidant contributors in selenium-sand melon juice. HPP maintained significantly higher scavenging activity throughout storage, consistent with Szczepańska et al. [35], who reported superior phenolic and antioxidant retention in HPP- versus heat-treated carrot juice. This likely reflects combined preservation of native antioxidants and pressure-enhanced phenolic extractability [36].

Although the superiority of HPP over thermal treatment for antioxidant retention is not a foregone conclusion in the literature—pressure effects on antioxidant activity during storage are product- and condition-dependent, with reports of increases, no change, or accelerated losses [15], and thermal processing can even raise measurable total phenolic content in some matrices by releasing cell-wall-bound phenolics [8], HPP alone in this study achieved comparable results without additives. This positions HPP as a highly effective single-step non-thermal technology, likely due to its simultaneous inactivation of oxidative enzymes, preservation of heat-labile antioxidants, and enhancement of phenolic extractability via cell wall disruption [7,36].

### 3.5. Effects of Different Treatments on Volatile Compounds in SSJ During Storage

GC-MS analysis identified 56 volatile organic compounds (VOCs) in SSJ during 35 days of storage, comprising 20 aldehydes, 17 alcohols, 8 ketones, 5 alkanes/olefins, 5 acids, 3 esters, and 2 furans (Table S1). Characteristic fresh, green, grassy notes—primarily from C6/C9 aldehydes (hexanal, nonanal) and alcohols—were significantly better preserved in HPP-treated groups than LTLT groups, consistent with our IEF study showing superior retention of fresh-character volatiles (nonanal, (E,E)-2,4-nonadienal, cis-6-nonen-1-ol) compared to pasteurization [17], and our UV-C study demonstrating broader volatile retention with antioxidant combination (12 vs. 9 key compounds in HTST) [18].

**Figure 6:**
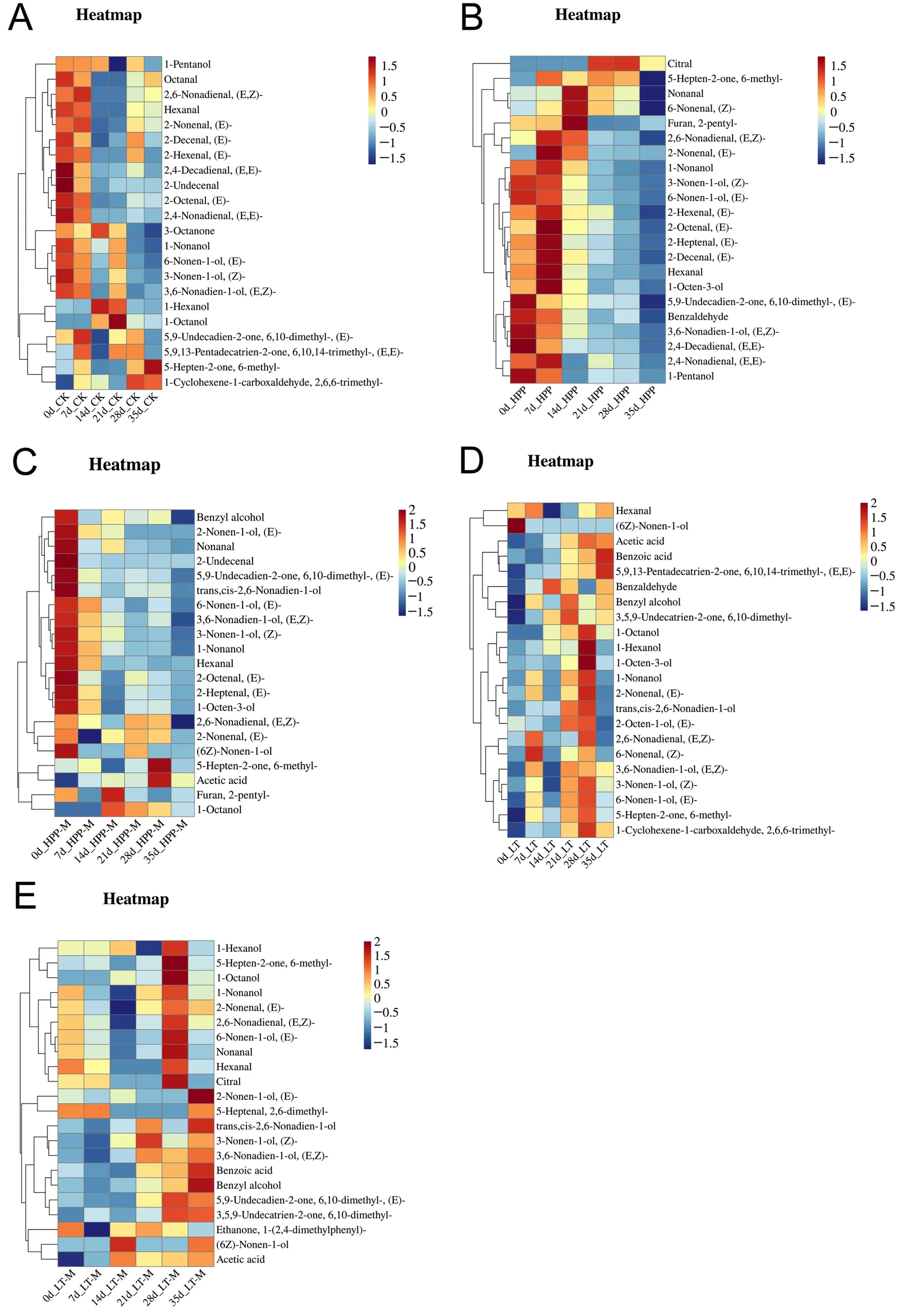
Heatmap visualization of volatile flavor profiles in SSJ across various treatments for 35-day storage: (**A**) CK (Control), (**B**) HPP, (**C**) HPP-M, (**D**) LTLT, (**E**) LTLT-M. Darker red shading indicates higher relative abundance, while darker blue shading indicates lower relative abundance.

Cluster heat maps (Figure 6A–E) revealed distinct volatile evolution patterns. In the CK group, fresh-characteristic compounds (3-nonen-1-ol, 3,6-nonadien-1-ol, 6-nonen-1-ol, 1-nonanol) declined sharply, with retention rates of only 23% (cis-3-nonen-1-ol) and 25% (6-nonen-1-ol) by day 35. Concurrently, oxidation markers accumulated substantially: 6-methyl-5-hepten-2-one increased 112%, and β-cyclocitral increased 204%, reflecting pronounced oxidative deterioration. In contrast, HPP and HPP-M groups retained significantly higher levels of characteristic C9 aldehydes and alcohols; 2-nonenal retention reached 96% in HPP by day 35 (versus 49% in CK), and 3-nonen-1-ol content remained 1.5-fold higher than CK. Notably, 6-methyl-5-hepten-2-one initially increased but declined below baseline by day 35 in HPP samples, indicating effective suppression of carotenoid oxidation [37]—consistent with our IEF findings showing gradual geranylacetone decline mitigated by treatment (2.59% retention at day 25) [17]. LTLT and LTLT-M groups showed pronounced acetic acid accumulation, likely from residual or sublethally injured microorganisms resuming metabolism [38], a recognized marker of microbial spoilage in thermally processed juices, particularly from Acetobacter species [38]. This parallels our UV-C study, where heat-treated samples accumulated off-flavor markers (toluene, styrene) indicative of thermal impact [18].

**Figure 7:**
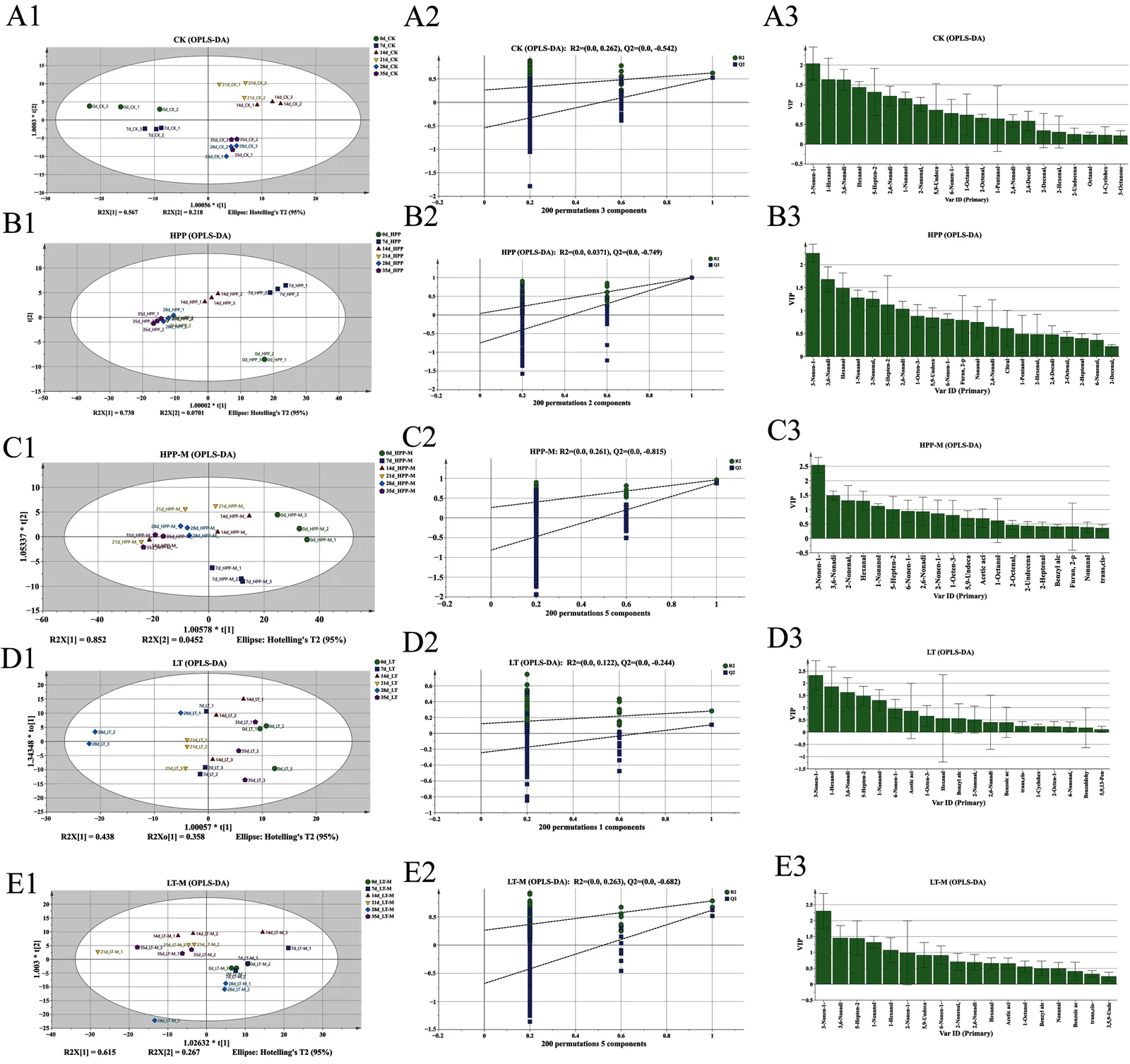
(1) OPLS-DA score plots, (2) permutation test validation, and (3) VIP chart of volatile compounds in SSJ subjected to various treatments over a 35-day storage period: (**A1**–**A3**) CK (Control), (**B1**–**B3**) HPP, (**C1**–**C3**) HPP-M, (**D1**–**D3**) LTLT, (**E1**–**E3**) LTLT-M. Model robustness was evaluated via 200-permutation tests for each treatment group.

OPLS-DA modeling further characterized these differences, with model validity confirmed via scatter plots and permutation tests (Figure 7A1–E1). All five models showed negative Q^2^ intercepts and R^2^Y intercepts below 0.3 (Control: R^2^Y = 0.262, Q^2^ = −0.542; HPP: R^2^Y = 0.037, Q^2^ = −0.749; HPP-M: R^2^Y = 0.261, Q^2^ = −0.815; LTLT: R^2^Y = 0.122, Q^2^ = −0.244; LTLT-M: R^2^Y = 0.263, Q^2^ = −0.682), indicating that the OPLS-DA models were valid and free from overfitting. The HPP model showed strong discrimination (t[1] = 73.8%, t[2] = 7.0%; cumulative 80.8%). VIP analysis (≥1, Figure 7A3–E3) combined with univariate one-way ANOVA (*p* < 0.05) and fold-change screening to reduce false positives associated with VIP alone, identified 3-nonen-1-ol and 3,6-nonadien-1-ol as key differentiating compounds across all groups (VIP 2.04–2.93 and 1.45–2.41, respectively), reflecting their importance to fresh flavor quality [9]. CK showed the most VIP ≥ 1 compounds (8), including both C6 (1-hexanol, hexanal) and C9 species, confirming substantial flavor deterioration. HPP retained 7 VIP ≥ 1 compounds, dominated by C9 aldehydes (2-nonenal, 2,6-nonadienal). Notably, HPP-M showed only 4 VIP ≥ 1 compounds, all C9 species (3-nonen-1-ol, 3,6-nonadien-1-ol, 1-nonanol, 2-nonenal), with no C6 compounds reaching threshold (hexanal VIP = 0.99)-demonstrating that HPP effectively inactivated inoculated microorganisms, limiting flavor evolution to natural C9 decline rather than microbial spoilage. This aligns with our UV-C study, where the HH group showed minimal flavor drift due to reduced thermal loss and antioxidant-mediated degradation control [18]. Conversely, LTLT showed 5 VIP ≥ 1 compounds, including 1-hexanol (VIP = 1.86) and 6-methyl-5-hepten-2-one (VIP = 1.48), indicating C6 alcohol accumulation; acetic acid’s near-threshold VIP (0.87) suggested microbial activity. LTLT-M similarly showed 5 VIP ≥ 1 compounds, with 6-methyl-5-hepten-2-one, 1-hexanol, and acetic acid near threshold. Principal component displacement was significantly greater for LTLT and LTLT-M than HPP groups (Figure 7D1,E1), confirming more severe flavor deterioration under thermal treatment. Our LTLT-treated SSJ samples did not exhibit a substantial buildup of furans and furanones as thermal degradation markers, in contrast to Cheng et al.’s [11] results that thermal processing of mandarin juice produced these chemicals. This could be a result of the milder LTLT circumstances (65 °C for 30 min) compared to the HTST conditions (85 °C for 15 s) investigated in that study, indicating that furan formation is primarily driven by temperature intensity rather than processing time. Nonetheless, the acetic acid accumulation seen in our LTLT groups—which was not noted in samples treated with HPP—aligns with the microbial regrowth patterns found in Section 3.1 and lends credence to the theory that residual microbial metabolism, as opposed to purely chemical oxidation, is what causes off-flavor development in thermally pasteurized SSJ during extended cold storage.

These findings demonstrate that HPP provides dual advantages in microbial safety and flavor retention, preserving characteristic C9 aldehyde/alcohol profiles while preventing accumulation of microbially and oxidatively derived off-flavors. This complements our prior work on UV-C and IEF technologies, positioning HPP as particularly advantageous for opaque juice matrices where optical methods face inherent limitations [16,17,18].

## 4. Conclusions

This study systematically compared the efficacy of HPP (500 MPa, 10 min) and conventional LTLT pasteurization (65 °C, 30 min) on the storage quality and flavor of selenium-sand melon juice. HPP treatment ensured robust microbial safety, preventing regrowth of *E. coli* and *A. sclerotioniger* throughout 35 days of refrigerated storage, whereas LTLT-treated samples exhibited regrowth after 7 days, indicating that HPP effectively inhibits the metabolic recovery of sublethally injured cells. Furthermore, HPP significantly outperformed LTLT in preserving physicochemical and nutritional quality, as demonstrated by superior color stability (ΔE ≤ 1.58), pH maintenance, and retention of lycopene, total phenolics, and antioxidant activity. GC-MS and OPLS-DA analyses of volatile profiles revealed that HPP effectively preserved characteristic C9 aldehydes and alcohols while preventing accumulation of off-flavor compounds, whereas LTLT-treated samples showed signs of oxidative and microbial degradation, notably acetic acid accumulation.

These findings establish HPP as a superior processing technology for selenium-sand melon juice, providing more effective microbial safety and quality preservation compared to LTLT pasteurization. Our study demonstrates that non-thermal technologies offer versatile and effective solutions for melon-based product preservation, with HPP emerging as particularly suitable for opaque juice matrices where optical-based technologies face limitations. The systematic approach employed here, combining microbiological challenge testing, comprehensive physicochemical analysis, and advanced chemometric evaluation of volatile compounds, provides a robust framework for evaluating non-thermal processing technologies in complex food matrices.

However, as this study did not include a formal sensory evaluation, the impact of HPP on the perceived organoleptic qualities of selenium-sand melon juice warrants further investigation to fully validate its commercial application. Future studies should address industrial-scale validation, extended storage trials, sensory evaluation with consumer panels, and cost–benefit analysis to facilitate commercial adoption. Additionally, the integration of HPP with other preservation hurdles (such as natural antimicrobials or antioxidants) may offer further quality enhancement, building on the synergistic effects we have previously demonstrated for UV-C and antioxidant combinations.

## Data Availability

The data presented in this study are original and were generated by the authors. The data are available from the corresponding author upon reasonable request.

## Supplementary Materials

The following supporting information can be downloaded at: https://www.mdpi.com/article/10.3390/foods15183292/s1, Table S1. Qualitative and quantitative results of 56 aromatic compounds determined by GC-MS.
