# Supplementary material for: Effect of High-Pressure Processing on Storage Quality, Bioactive Compounds, and Flavor Profile of Selenium-Sand Melon Juice
Source: Foods. 2026 Sep 17;15(18):3292. doi: 10.3390/foods15183292 (PMC13606920; doi:10.3390/foods15183292)
Supplement: Supplementary file 1 [file foods-15-03292-s001.zip › foods-4526141-supplementary.pdf]

Supplementary Material

Table S1. Qualitative and quantitative results of 56 aromatic compounds determined by GC-MS

	Volatile compound content (μg/mL)																													
**Compound name**	**0d CK**	**0d LTLT**	**0d LTLT-M**	**0d HPP**	**0d HPP-M**	**7d CK**	**7d LTLT**	**7d LTLT-M**	**7d HPP**	**7d HPP-M**	**14d CK**	**14d LTLT**	**14d LTLT-M**	**14d HPP**	**14d HPP-M**	**21d CK**	**21d LTLT**	**21d LTLT-M**	**21d HPP**	**21d HPP-M**	**28d CK**	**28d LTLT**	**28d LTLT-M**	**28d HPP**	**28d HPP-M**	**35d CK**	**35d LTLT**	**35d LTLT-M**	**35d HPP**	**35d HPP-M**
Benzaldehyde	1.5806	1.8339	1.7666	5.3302	3.1012	1.7945	3.3814	1.3035	4.4730	1.4432	0.1178	5.2188	0.0000	2.3803	1.0016	1.1917	4.2394	1.5373	1.2141	1.0946	2.7649	2.2965	3.7556	2.0283	1.3145	1.4154	4.3730	1.7857	0.0000	0.7003
Octanal	1.3591	0.7059	0.8348	1.9005	1.7776	0.9894	0.9444	0.5905	1.8996	0.0000	0.0000	0.0000	0.0000	1.1582	0.0000	0.0000	0.5154	0.0000	0.6973	0.0000	0.5750	1.0106	0.9825	0.0000	0.0000	0.8644	0.4555	0.0000	0.5156	0.4941
Nonanal	8.5729	3.2647	5.4830	8.7176	11.6967	8.5504	4.3920	3.5504	8.8902	5.3165	0.3616	3.8253	0.1146	14.0088	6.9208	0.6579	3.4173	2.9481	10.4187	4.5651	5.4024	3.7539	9.8279	9.2001	4.2067	5.3037	2.7653	2.1609	4.3630	3.4922
2-Heptenal, (E)-	6.6608	1.7341	1.2481	6.4267	6.0425	4.7120	2.5216	0.7287	8.6394	3.5311	0.0000	0.6846	0.0000	4.4265	1.2597	0.0000	0.9925	0.8662	3.7486	2.6839	2.2229	2.7340	2.2909	2.8927	2.4737	1.7909	1.9162	0.4670	1.9134	1.7328
2-Nonenal, (E)-	22.48	4.671	10.37	16.95	35.74	25.0	7.468	7.149	45.37	2.778	0.69	4.010	0.652	35.33	26.82	1.99	6.898	9.534	18.1	31.62	14.5	9.859	13.53	17.2	29.87	10.9	3.633	10.97	16.2	16.32

	66	1	80	25	87	892	5	4	61	6	31	8	5	39	94	60	4	7	285	39	294	6	49	320	31	169	0	80	653	30
6-Nonenal, (Z)-	0.5752	0.6879	1.5691	1.8491	1.0359	0.8926	1.5572	0.8262	2.1867	1.2366	0.0197	0.6619	0.0000	3.2493	0.6776	0.0000	1.0331	0.4136	2.3717	0.6136	1.0018	1.2687	2.3394	2.0104	0.9752	1.2022	0.7102	0.4230	0.7914	0.3842
2-Undecenal	2.0000	0.4451	0.3248	0.0000	3.8774	1.0118	0.0000	0.0000	1.6498	0.3798	0.0000	0.0000	0.0000	0.0000	0.1849	0.2842	0.0000	0.0000	0.6672	0.1775	0.2850	0.4674	0.0000	0.2765	0.2360	0.2554	0.6050	0.0000	0.1979	0.2203
2,4-Decadienal, (E,E)-	8.4160	0.0000	2.8225	10.6588	6.5626	4.2091	0.0000	0.0000	6.5271	0.0000	0.2185	1.0070	0.0000	3.1658	0.0000	0.4635	2.4331	0.0000	2.2364	0.0000	2.2620	6.2305	2.7887	2.1738	0.0000	0.0000	0.0000	1.6060	0.0000	0.0000
2-Octenal, (E)-	10.7416	1.7079	1.4134	5.9950	6.5873	8.9597	2.2466	1.0053	9.3874	3.0131	0.2061	0.9264	0.0000	5.4731	1.4131	0.5327	1.3103	0.5749	4.8231	3.2252	3.7388	2.5539	2.5912	3.8855	2.6555	3.1166	1.7947	0.5174	2.6073	1.9401
2-Decenal, (E)-	3.3627	0.6449	0.5975	1.9037	3.9489	2.3254	0.5027	0.2329	2.6602	0.6101	0.1214	0.4156	0.0000	1.6071	0.2696	0.7416	0.5780	0.0000	1.3006	0.8302	2.6201	1.0755	0.7268	1.0143	0.5554	1.0771	0.5123	0.0000	0.6693	0.4868
1-Cyclohexene-1-carboxaldehyde, 2,6,6-trimethyl-	0.4106	0.4204	0.4154	1.0404	0.9117	0.9501	0.9510	0.3649	0.8106	0.8424	0.8601	0.8198	0.2562	0.7726	0.5888	0.6029	1.3202	0.6868	0.9795	0.8508	1.3199	1.8128	1.1782	0.9281	1.1432	1.2492	1.3168	0.6412	0.4238	0.6672
2,6-Nonadienal, (E,Z)-	28.8527	9.4980	11.2676	28.8236	29.9096	35.126	16.5573	8.0864	41.9150	25.6140	0.0000	8.6109	0.7250	38.1929	21.2078	0.0000	9.6160	7.6445	23.597	28.9334	13.730	17.4126	16.1938	22.016	28.2987	16.176	6.5236	8.8801	14.878	14.1540

2,4-Nonadienal, (E,E)-	7.7474	2.0481	2.1967	8.8063	1.1576	55.3751	1.9389	0.4841	10.6715	0.0000	0.0000	0.5584	0.0000	2.1290	1.7767	0.0000	0.0000	0.0000	55.0634	0.0000	41.2579	4.0718	0.0000	53.8251	0.0000	10.2738	0.0000	0.0000	31.8281	3.4147
Hexanal	48.7113	14.7448	10.7574	67.8546	74.4200	43.4623	18.0543	6.3889	97.2747	46.9274	0.0000	0.6108	0.0000	43.8746	17.8853	0.0000	6.8304	0.0000	26.0977	21.0251	23.9595	13.0092	12.9296	22.6215	15.6568	21.3140	16.1873	4.1839	15.5012	17.1366
2-Hexenal, (E)-	2.3696	0.4804	0.0000	2.3230	1.4620	2.1064	0.9591	0.0000	3.0449	0.0000	0.0000	0.0000	0.0000	1.6329	0.0000	0.0000	0.0000	0.0000	1.4567	1.2001	1.6867	0.0000	0.0000	0.6001	1.1166	0.0000	0.0000	0.0000	0.0000	0.7139
3-Octanone	0.9220	0.4663	0.0000	1.4056	0.0000	0.7893	0.8658	0.2805	0.0000	0.0000	1.0435	0.0000	0.0000	0.0000	0.0000	0.8246	0.0000	0.0000	0.0000	0.0000	0.4678	1.0835	0.0000	0.0000	0.0000	0.3643	0.0000	0.0000	0.0000	0.0000
5-Hepten-2-one, 6-methyl-	13.7081	18.5844	28.7861	37.2861	28.9392	19.2905	56.2296	32.2029	55.9838	30.2751	9.3150	45.2543	14.5098	48.4050	21.8810	9.7191	66.5809	31.5374	54.3566	28.4600	20.4741	76.7550	85.1944	51.7487	41.5938	29.0014	48.2433	30.7439	27.8854	26.5830
5,9,13-Pentadecatrien-2-one, 6,10,14-trimethyl-, (E,E)-	0.5359	0.2065	0.1557	0.9930	0.4571	0.9624	0.5650	0.2199	0.5222	0.3832	0.2694	0.6222	0.1731	0.2824	0.0000	0.8922	0.8369	0.3900	0.3312	0.3727	0.8905	0.9140	0.3751	0.2943	0.5149	0.4013	1.3186	0.2998	0.2559	0.1794
trans-3-Nonen-2-one	0.428	0.146	0.000	0.712	0.000	0.44	0.000	0.000	0.382	0.000	0.09	0.000	0.000	0.000	0.000	0.03	0.000	0.000	0.21	0.000	0.26	0.516	0.000	0.26	0.000	0.25	0.000	0.000	0.00	0.000

	3	2	0	0	0	67	0	0	5	0	55	0	0	0	0	87	0	0	12	0	61	7	0	94	0	63	0	0	00	0
5,9-Undecadien-2-one, 6,10-dimethyl-, (E)-	19.1311	12.7560	10.4431	38.7173	40.4409	23.3157	21.8550	7.7388	27.0128	22.9682	11.8673	20.5093	6.9844	23.7103	19.3324	18.3176	30.6998	16.2000	20.6080	21.1319	20.2740	28.4915	24.9435	19.9387	19.9951	13.9422	17.4972	22.1660	8.3921	12.7327
Ethanone, 1-(2,4-dimethylphenyl)-	0.6433	0.5740	0.7024	0.0000	0.0000	0.0000	0.0000	0.0000	0.0000	0.0000	0.0000	0.4202	0.5126	0.0000	0.6160	0.0000	0.0000	0.6444	0.2885	0.5520	0.0000	0.6404	0.5010	0.3970	0.4665	0.1505	0.3309	0.3342	0.2378	0.0000
Furan, 2-pentyl-	1.9874	1.0985	2.0449	3.7809	4.6044	2.9270	0.9755	1.6136	3.9600	0.0000	0.7922	0.0000	0.0000	6.4486	6.8018	0.7556	0.0000	0.0000	1.2448	1.4849	3.2399	0.0000	2.6196	1.3142	0.0000	1.3889	0.0000	0.0000	2.1881	1.5689
Octanoic acid, methyl ester	0.3919	0.0000	0.0000	0.0000	0.0000	0.3085	0.0000	0.0000	0.0000	0.0000	0.0000	0.3126	0.0000	1.0786	0.0000	0.0000	0.5834	0.0000	2.3472	0.0000	0.0000	1.4823	0.0000	2.1560	0.6761	0.5236	0.4155	0.0000	0.9145	0.0000
Hexadecanoic acid, methyl ester	0.2803	0.0000	0.0000	0.0000	0.0000	1.2835	0.0000	0.0000	0.0000	0.0000	0.0000	0.0000	0.0000	0.0000	0.0000	0.0000	0.0000	0.0000	0.0000	4.7135	0.0000	0.0000	0.0000	0.0000	0.0000	0.0000	0.0000	0.0000	0.0000	0.0000
Benzyl alcohol	4.6715	5.4500	3.5130	11.1973	9.6130	4.7441	8.6660	2.6834	6.5542	6.1432	0.0000	7.3097	3.6026	4.9848	7.2167	1.5268	9.9321	4.9076	4.7737	6.6080	5.0430	8.1677	6.0079	4.3616	6.9535	4.0780	9.0532	8.7648	2.1077	4.3266
3-Nonen-1-ol, (Z)-	124.8	149.8	163.2	264.2	681.3	95.4	183.0	122.7	252.8	439.3	37.2	131.2	230.1	148.1	365.2	74.5	204.5	334.3	83.5	242.1	36.5	221.5	217.0	65.9	258.0	28.9	177.5	285.0	43.1	142.9

	126	676	774	523	504	138	340	707	248	148	464	820	443	846	999	811	861	647	094	112	151	503	089	708	959	674	925	719	323	162
1-Hexanol	8.3369	7.0609	7.4113	18.7876	0.0000	6.9349	17.6429	7.5140	17.1476	11.1747	45.8438	28.2518	9.6151	10.5673	0.0000	38.8317	24.2676	0.0000	4.1964	7.3280	7.1448	71.1193	14.2289	5.4941	10.5818	5.6776	9.9275	5.5666	3.4405	3.7019
1-Nonanol	33.0628	38.0352	64.7726	71.0871	125.3990	25.5418	56.1996	47.2754	84.7050	88.0930	16.8737	37.6550	34.6443	53.8212	58.4126	25.6813	61.0317	60.7283	28.9367	43.2744	8.4887	71.8516	74.5071	20.6333	45.3040	5.8667	37.7813	55.0579	14.3282	23.9634
2-Octen-1-ol, (E)-	1.7090	1.2487	0.5211	2.2051	2.4313	1.1456	1.1158	0.5122	2.5516	0.0000	0.7659	0.8513	0.0000	1.2722	0.0000	0.4179	1.9775	0.0000	0.8304	0.7294	0.7882	2.0825	0.4320	0.5940	1.1850	0.6486	0.5970	0.0000	0.4639	0.7082
trans,cis-2,6-Nonadien-1-ol	1.6190	2.5638	1.3550	4.7309	6.6556	1.7908	2.8702	0.6915	3.4098	2.9103	1.3176	2.8436	1.7461	2.0667	2.2265	2.0439	3.5310	3.2879	0.7237	2.9553	0.8141	3.7220	1.5808	0.6240	2.9802	0.5372	2.5341	4.0843	0.6550	1.4802
2-Nonen-1-ol, (E)-	3.8452	4.3995	2.8862	8.0915	25.6603	2.5699	5.9922	0.9424	0.0000	10.9493	3.1682	2.2308	3.3144	0.3339	7.5790	5.2767	0.0000	0.0000	0.0000	0.0000	0.0000	0.0000	0.0000	0.0000	0.0000	0.0000	0.0000	14.0602	0.0000	0.0000
6-Nonen-1-ol, (E)-	14.9146	17.4352	23.1231	36.5684	32.9854	12.8223	29.5885	17.6346	33.6524	26.0553	6.3974	20.0671	10.2776	19.4520	15.2506	12.2611	33.5078	15.4537	12.2207	9.1272	5.0636	37.4145	32.3073	9.9155	15.6822	3.7505	25.6943	16.6822	5.9560	7.5459
1-Octen-3-ol	7.9292	5.3902	4.7523	14.5762	15.8227	7.2404	6.2175	3.5667	22.3247	9.7466	4.3213	5.4331	0.0000	11.4803	2.0803	7.7693	8.4770	0.0000	6.5149	6.5201	9.6993	14.9817	5.8520	8.1253	6.6825	5.2456	6.8379	0.0000	4.0794	3.7943

3,6-Nonadien-1-ol, (E,Z)-	65.9396	87.8279	71.0600	157.2986	231.1877	60.7900	124.8833	54.3855	119.9009	2.2645	23.9411	84.9197	82.6577	77.3738	138.6095	51.8741	128.3334	120.7800	52.8428	109.0886	22.2818	124.0030	110.7420	46.6465	136.2988	27.9321	114.8600	125.0291	26.8576	69.0864
1-Pentanol	5.3208	0.0000	0.0000	7.2971	0.0000	5.2879	5.6409	0.0000	5.2963	0.0000	5.0102	1.9710	0.0000	0.0000	0.0000	0.0000	0.0000	0.0000	1.7727	0.0000	4.5123	6.6599	0.0000	1.6172	0.0000	1.9444	0.0000	0.0000	0.0000	0.0000
Benzoic acid	0.6640	1.0706	1.1300	0.0000	2.6419	0.7322	0.6672	0.3132	0.0000	0.0000	0.0000	1.3250	0.0000	0.0000	3.7900	0.0000	2.2854	1.9905	0.0000	0.0000	0.0000	2.8038	2.4139	0.0000	0.0000	0.0000	3.9632	3.7056	0.0000	0.0000
(6Z)-Nonen-1-ol	0.0000	28.5528	0.0000	0.0000	34.1272	0.0000	0.8739	0.0000	0.0000	0.0000	10.7683	0.0000	15.9197	0.0000	0.0000	0.0000	0.0000	0.0000	0.0000	18.7211	0.0000	0.0000	0.0000	0.0000	0.0000	0.0000	0.0000	12.1689	0.0000	0.0000
5-Heptenal, 2,6-dimethyl-	0.0000	0.0000	0.4526	0.0000	0.0000	0.2980	0.6493	0.4602	0.0000	0.0000	0.0000	0.5139	0.0000	0.6427	0.0000	0.0000	0.0000	0.0000	0.6341	0.2311	0.3970	0.0000	0.0000	0.6329	0.0000	0.2850	0.0000	0.4580	0.3304	0.4244
Acetophenone	0.0000	0.4909	0.4994	0.0000	0.0000	0.0000	0.0000	0.0000	0.3346	0.0000	0.0000	0.3094	0.0000	0.3546	0.0000	0.0000	0.6173	0.0000	0.0000	0.0000	0.0000	0.4535	0.8423	0.0000	0.0000	0.0000	0.4886	0.0000	0.2632	0.0000
3,5,9-Undecatrien-2-one, 6,10-dimethyl-	0.0000	0.0000	0.0000	0.0000	0.0000	0.2696	0.3493	0.3321	0.4113	0.0000	0.0722	0.6743	0.0836	0.2921	0.0000	0.1776	0.9530	0.3544	0.6144	0.1590	0.8781	0.5432	1.0237	0.7581	0.4867	0.3445	0.6406	0.9654	0.5550	0.2252

Citral	0.0000	0.0000	4.3354	0.0000	0.0000	2.9719	5.1478	4.7486	0.0000	0.0000	0.0000	0.0000	0.0000	0.0000	0.0000	0.0000	0.0000	0.0000	7.8975	0.0000	3.9595	0.0000	10.7345	7.9726	0.0000	2.7448	0.0000	0.0000	3.6716	0.0000
1-Dodecanol	0.1076	0.0000	0.0000	0.0000	0.0000	0.0745	0.0000	0.0000	0.2507	0.0000	0.0610	0.0876	0.0000	0.0000	0.0000	0.0000	0.0000	0.0000	0.0856	0.0928	0.0781	0.0000	0.0000	0.0000	0.4068	0.1577	0.4316	0.2143	0.0992	0.1678
Octanoic acid	0.0000	0.0000	0.0000	0.0000	0.0000	0.1926	0.0000	0.1344	0.7111	0.0000	0.0000	0.0000	0.0000	0.3896	0.0000	0.0000	0.0000	0.0000	0.5070	0.0000	0.7056	0.0000	0.0000	0.5023	0.0000	0.2733	0.0000	0.0000	0.4921	0.0000
Decanal	0.0000	0.5698	0.1863	0.0000	1.9627	0.0000	0.2316	0.0395	0.0000	0.0000	0.0469	0.1468	0.0000	0.0000	0.0000	0.0000	0.0000	0.0000	0.0000	0.0000	0.0000	0.0000	0.4583	0.0000	0.0000	0.3324	0.0000	0.4278	0.0000	0.0000
Acetic acid	0.0000	0.6380	0.0000	1.2452	0.0000	0.0000	1.7911	3.0283	5.9356	4.7586	4.1195	4.8442	8.3168	4.3308	5.5209	5.8034	7.4919	5.9587	5.1769	5.1011	4.8250	10.3446	6.6620	4.1014	11.9193	4.0906	9.5475	7.8059	4.4313	5.7946
Octadecane	0.0000	0.0000	0.0000	0.0000	0.0000	0.0000	0.6455	0.0000	0.0000	0.0000	0.0000	0.0000	0.0000	0.0000	0.0000	0.0000	0.0000	0.0000	0.0670	0.0000	0.0000	0.0000	0.0000	0.0000	0.0000	0.0000	0.0000	0.0000	0.0000	0.0000
Tetradecane	0.0000	0.0000	0.0000	0.0000	0.0000	0.0000	0.3693	0.3052	0.0000	3.2268	0.9281	0.7480	1.1300	0.0000	1.0370	0.7117	0.0000	1.0925	1.0898	3.3192	0.0000	0.0000	0.0000	0.0000	4.0691	0.0000	0.0000	0.0000	0.2615	0.4554
Pentadecane	0.0000	0.0000	0.0000	0.0000	0.0000	0.0000	0.5162	0.0000	0.0000	0.0000	0.0000	0.6980	0.0000	0.0000	0.9608	0.0000	0.0000	1.0850	0.0000	0.0000	0.0000	0.0000	0.0000	0.4784	0.8675	0.0000	0.0000	0.0000	0.0000	0.0000
Hexadecane	0.000	0.000	0.000	0.000	0.000	0.00	0.000	0.155	0.000	0.000	0.55	0.000	0.000	0.000	0.000	0.39	1.014	0.766	0.00	0.000	0.60	3.965	0.926	0.00	0.000	0.00	0.000	0.000	0.00	0.000

	0	0	0	0	0	00	0	7	0	0	33	0	0	0	0	38	6	2	00	0	86	8	7	00	0	00	0	0	00	0
Citronellol	0.0000	0.0000	0.0000	0.0000	0.0000	0.0000	0.0000	0.0000	0.0000	0.0000	0.0400	0.0000	0.0000	0.0000	0.0000	0.0000	0.0000	0.0000	0.0000	0.0000	0.0000	0.0000	0.0000	0.0000	0.0000	0.0000	3.0394	0.0000	0.0000	0.0000
1-Octanol	0.0000	0.0000	0.0000	0.0000	0.0000	0.0000	0.0000	0.0000	0.0000	0.0000	4.9869	5.6287	1.8233	5.5343	3.8520	8.9222	7.7800	1.2906	0.0000	3.1332	2.3691	12.7882	6.5336	2.6017	2.4868	1.6370	5.2438	1.5624	1.8435	1.3536
n-Tridecan-1-ol	0.0000	0.0000	0.0000	0.0000	0.0000	0.0000	0.0000	0.0000	0.0000	0.5174	0.0000	0.0000	0.0000	0.0000	0.0000	0.0000	0.2500	0.0000	0.0000	0.0000	0.2580	0.0000	0.0000	0.0000	0.2570	0.0000	0.0000	0.0000	0.0000	0.3628
Pentanal	0.0000	0.0000	0.0000	1.2855	0.0000	0.0000	0.0000	0.0000	0.0000	0.0000	0.0000	0.0000	0.0000	0.0000	0.0000	0.0000	0.0000	0.0000	0.6784	0.0000	0.1855	0.0000	0.0000	0.0000	0.0000	0.4824	0.0000	0.0000	0.0000	0.0000
7-Tetradecene, (E)-	0.0000	0.0000	0.0000	0.0000	0.0000	0.0000	0.0000	0.0000	0.0000	0.0000	0.0000	0.0000	0.0000	0.0000	0.0000	0.0000	0.0000	0.0000	0.0000	0.3811	1.6233	0.0000	0.0000	0.0000	0.0000	0.0000	0.0000	0.0000	0.3332	0.5382
Butanal, 2-methyl-	0.0000	0.0000	0.0000	0.0000	0.0000	0.0000	0.0000	0.0000	0.0000	0.0000	0.0000	0.0000	0.0000	0.0000	0.0000	0.0000	0.0000	0.0000	0.0000	0.0000	0.0000	0.0000	0.0000	0.0000	0.0000	0.2990	0.0000	0.0000	0.0000	0.0000
1-Tetradecanol	0.0000	0.0000	0.0000	0.0000	0.0000	0.0000	0.0000	0.0000	0.0000	0.0000	0.0000	0.0000	0.0000	0.0000	0.0000	0.0000	0.0000	0.0000	0.0000	0.0000	0.0000	0.0000	0.0000	0.0000	0.0000	0.0000	0.0000	0.0000	0.0000	0.4272
